# Effect of a Functional Phospholipid Metabolome-Protein Association Pathway on the Mechanism of COVID-19 Disease Progression

**DOI:** 10.7150/ijbs.72450

**Published:** 2022-07-11

**Authors:** Mingshan Xue, Teng Zhang, Zhangkai J. Cheng, Baojun Guo, Yifeng Zeng, Runpei Lin, Peiyan Zheng, Mingtao Liu, Fengyu Hu, Feng Li, Wensheng Zhang, Lu Li, Qi Zhao, Baoqing Sun, Xiaoping Tang

**Affiliations:** 1 Guangzhou Eighth People's Hospital, Guangzhou Medical University, Guangzhou, 510060, China.; 2 Guangzhou Laboratory, XingDaoHuanBei Road, Guangzhou International Bio Island, Guangzhou 510005, Guangdong Province, China.; 3 National Center for Respiratory Medicine, The First Affiliated Hospital of Guangzhou Medical University, National Clinical Research Center for Respiratory Disease, State Key Laboratory of Respiratory Disease, Guangzhou Institute of Respiratory Health, Guangzhou 510120, China.; 4 MoE Frontiers Science Center for Precision Oncology, Cancer Centre, Institute of Translational Medicine, Faculty of Health Sciences, University of Macau. Taipa, Macau, China.; 5 Institue of automation Chinese Academy of Sciences, Beijing, China.

**Keywords:** COVID-19, phospholipid metabolic pathway, eicosanoic acids, metabolomics

## Abstract

This study aimed to explore the clinical practice of phospholipid metabolic pathways in COVID-19. In this study, 48 COVID-19 patients and 17 healthy controls were included. Patients were divided into mild (n=40) and severe (n=8) according to their severity. Phospholipid metabolites, TCA circulating metabolites, eicosanoid metabolites, and closely associated enzymes and transfer proteins were detected in the plasma of all individuals using metabolomics and proteomics assays, respectively. 30 of the 33 metabolites found differed significantly (*P*<0.05) between patients and healthy controls (*P*<0.05), with D-dimmer significantly correlated with all of the lysophospholipid metabolites (LysoPE, LysoPC, LysoPI and LPA). In particular, we found that phosphatidylinositol (PI) and phosphatidylcholine (PC) could identify patients from healthy controls (AUC 0.771 and 0.745, respectively) and that the severity of the patients could be determined (AUC 0.663 and 0.809, respectively). The last measurement before discharge also revealed significant changes in both PI and PC. For the first time, our study explores the significance of the phospholipid metabolic system in COVID-19 patients. Based on molecular pathway mechanisms, three important phospholipid pathways related to Ceramide-Malate acid (Cer-SM), Lysophospholipid (LPs), and membrane function were established. Clinical values discovered included the role of Cer in maintaining the inflammatory internal environment, the modulation of procoagulant LPA by upstream fibrinolytic metabolites, and the role of PI and PC in predicting disease aggravation.

## 1. Introduction

One of the main pulmonary pathological symptoms of COVID-19 was varying degrees of bilateral lung involvement 1. Diffuse alveolar damage or interstitial lung injury may occur in some patients, which further affecting lung ventilation, especially in severe patients 1, 2. COVID-19 was similar to common influenza in terms of lung manifestations, but differed in terms of vascular involvement, with SARS-CoV-2 viruses more likely to trigger microvascular effects such as exudation, dilation, and embolization 1. Lung histopathology in COVID-19 patients revealed abnormal effects such as remodeling of extracellular mechanisms and epithelial proliferation, which correlated with disease severity and progression 3. The invasion of SARS-CoV-2 triggered an over-activation of the immune system, resulting in the formation of an inflammatory storm and causing multi-organ failure 4. Although this was a well-recognized trend of acute exacerbations, the inflammatory cells and factors commonly used in clinical practice cannot fully reflect the patient's condition 5. Therefore, most studies related to immune dysfunction have focused on severe patients, as extreme alterations in the homeostasis of the body's internal environment cause more significant changes in indicators 6.

Metabolomics and proteomics are sensitive trace analysis methods that allow rapid and simultaneous detection of multiple substances by applying small volumes of bodily fluids 7. They were more commonly used in molecular research and had advantages in analyzing the microenvironment of the organism 8. It could provide more detailed mechanistic information. Since we found significant changes in phospholipid-associated metabolites in our study, and Delafiori et al also reported abnormal manifestations of phospholipid metabolites during the non-targeted screening of metabolites 9, 10, this study will focus on using targeted analysis to explore this intriguing and unique change in the phospholipid pathway in COVID-19 patients.

Phospholipids were mainly divided into glycerophosphates and sphingolipids 11. The former contains phosphatidylcholine (lecithin), phosphatidylethanolamine (ceruloplasmin), and phosphatidylinositol 12. The latter was dominated by phosphoramides 12. Large molecules such as channel proteins, transporter proteins, and transferases play a regulatory role in controlling concentration gradients and maintaining normal life activities, forming a complete phosphate metabolic pathway with corresponding phospholipid metabolites 13. The classification of phospholipid metabolism was complex and variable, and it was the core link of many important processes such as physiological state, signal transduction, thrombosis, endothelial barrier, and regulation of inflammation 14. Therefore, it was reasonable to hypothesize that the SARS-CoV-2 virus disrupts the immune microenvironment of the body and then phospholipid metabolism also undergoes some alterations thus triggering lung damage effects. The systemic inflammatory effect of COVID-19 affects the respiratory system and could also involve the thyroid glands and multiple extrapulmonary organs such as the liver and kidneys 15, 16. Due to the wide range of functions of phospholipid metabolic pathways, the effects of inflammation, clotting, and stress are energy-consuming processes. Therefore, a combined TCA pathway and inflammatory pathway analysis reflect energy metabolism. Thus, in this research, the eicosanoid pathway (n-3/n-6), which reflects inflammation, and the TCA metabolic pathway of the tricarboxylic acid cycle, which reflects metabolic activity, were also involved and evaluated in conjunction with phospholipid metabolism to determine the impact of phospholipid metabolic pathways in disease.

## 2. Methods

### 2.1 Participant involvement

A total of 65 subjects, including COVID-19 patients (n=48) and healthy controls (n=17), from Guangzhou Eighth People's Hospital from 2020-01 to 2021-07 were included in the study. COVID-19 patients were diagnosed by positive SARS-CoV-2 PCR test, and whole-genome sequencing was used to identify delta variants. Patients were divided into mild and severe according to the severity of the disease. All patients had no previous hepatic or renal dysfunction or hematologic disease. On admission and before discharge, routine blood, liver and kidney function, and blood gases were obtained from each patient. The study was approved by Guangzhou Eighth People's Hospital Ethics Committee (No. 202001134 and 202115202). Written informed consents were obtained from all patients.

### 2.2 Severity criteria

Pulmonary-related indicators were used as the main assessment component in the study. Mild/general: mild clinical symptoms, no or little pneumonia visible on imaging. Severe/critical: significant respiratory symptoms (cough, shortness of breath, chest tightness, dyspnea); systemic manifestations such as fever, muscle pain; PaO2/FiO2 ≤ 300 mmHg; the need for mechanical ventilation; shock; multiple organ dysfunction syndromes (Including extensive damage to the vascular system, heart, kidneys and other organs 17); lung imaging suggesting significant large focal shadows (We refer to the pulmonary involvement scoring system of the Pan et al 18 study. 0, no involvement; 1, < 5% involvement; 2, 5-25% involvement; 3, 26-50% involvement; 4, 51-75% involvement; and 5, > 75% involvement. Mild: 1-3, severe: 4-5); other acute-phase symptoms, signs or instrumental indices.

### 2.3 Plasma collection

Because exercise affected the overall metabolic state, patients were at rest in the study. The individuals maintained sitting or recumbent position, did not exercise vigorously within 30 minutes, and did not drink sympathetic excitatory drinks.

Blood samples from the patient's first test after hospitalization and the last test before admission were collected and used for metabolic testing. Fasting blood samples were collected from subjects in the early morning and centrifuged at 3000 rpm for 10 minutes at room temperature within 2 hours on the same day. After collection, the plasmas were divided and stored in isolation at -80°C.

### 2.4 Sample preprocessing

Hydrophilic substance extraction: The plasma samples were thawed before testing. The proteins were then precipitated by adding 300 μl of pre-chilled methanol to 50 μl aliquots of plasma samples. After vortex-blending for 3 minutes and centrifuged at 12000 rpm for 10 minutes at 4°C, the 200 ul of supernatant was stood for 30 min at -20 °C. The extracts were centrifuged again at 12000 rpm for 3 minutes at 4°C and 150 ul of supernatant were collected for detection.

Hydrophobic substance extraction: Before testing, the plasma samples were thawed and centrifuged at 3000 rpm for 5 minutes at 4°C. 50 ul of samples were mixed with 1 ml of lipid extract (methyl tert-butyl ether: methanol = 3:1, with marker mixture). After vortex-blending for 15 minutes, the mixture was added to 200 μl of diluent water. Then the mixture was centrifuged again at 12000 rpm for 10 minutes at 4°C after vortex-blending for 15 minutes. 500 μl of supernatant was dried under a stream of N2 until all the extraction solvent was evaporated. The residue was resuspended in 200 µl of mobile phase (acetonitrile-0.1% formic acid) and transferred to detection.

### 2.5 Metabolomics and Proteomics detection

The study used ultraperformance liquid chromatography-mass spectrometry and tandem mass spectrometry (Wuhan Metware Biotechnology Co., Ltd, China) to analyze plasma samples with relatively quantitative targeted detection. Chromatographic column specifications: Thermo Accutancore C30, i.d. 2.1×100 mm, 2.6 μm. The flow rate of hydrophilic substance was 0.4 ml/min and the column temperature was 40℃. The flow rate of the hydrophobic substance was 0.35 ml/min and the column temperature was 45℃.

### 2.6 Statistics analysis

Continuous variables in this study were expressed using the median (interquartile range [IQR]) and categorical variables were expressed using number (frequency). Differences in continuous variables were tested using the Mann-Whitney-Wilcoxon rank-sum test (two groups) or the Kruskal-Wallis test (three or more groups). Categorical data were compared using the chi-square test (two groups) or Fisher's exact test (three or more groups). Data correlation analysis was performed using Spearman's correlation coefficient. Receiver operating characteristic (ROC) analysis was used to assess the predictive performance. Orthogonal Projections to Latent Structures Discriminant Analysis (OPLS-DA) was conducted to screen metabolites. The results of the analysis were considered statistically significant when *P* < 0.05. All statistical analyses in this study were performed using R software version 4.0.0 (R Core Team).

## 3. Results

### 3.1 Participants characteristic

The CRP and SAA in COVID-19 patients were higher than the normal range (10 mg/L and 10 mg/L, respectively). Lymphocyte (LYM) counts and LYM% were significantly lower in COVID-19 patients than in normal subjects, while monocyte (MONO) counts and MONO% were significantly higher in COVID-19 patients. The COVID-19 patients were further divided into mild (n=40) and severe (n=8).

### 3.2 Trend analysis for the metabolisms of a phospholipid, TCA cycle, and eicosanoic acids

**Figure 1:**
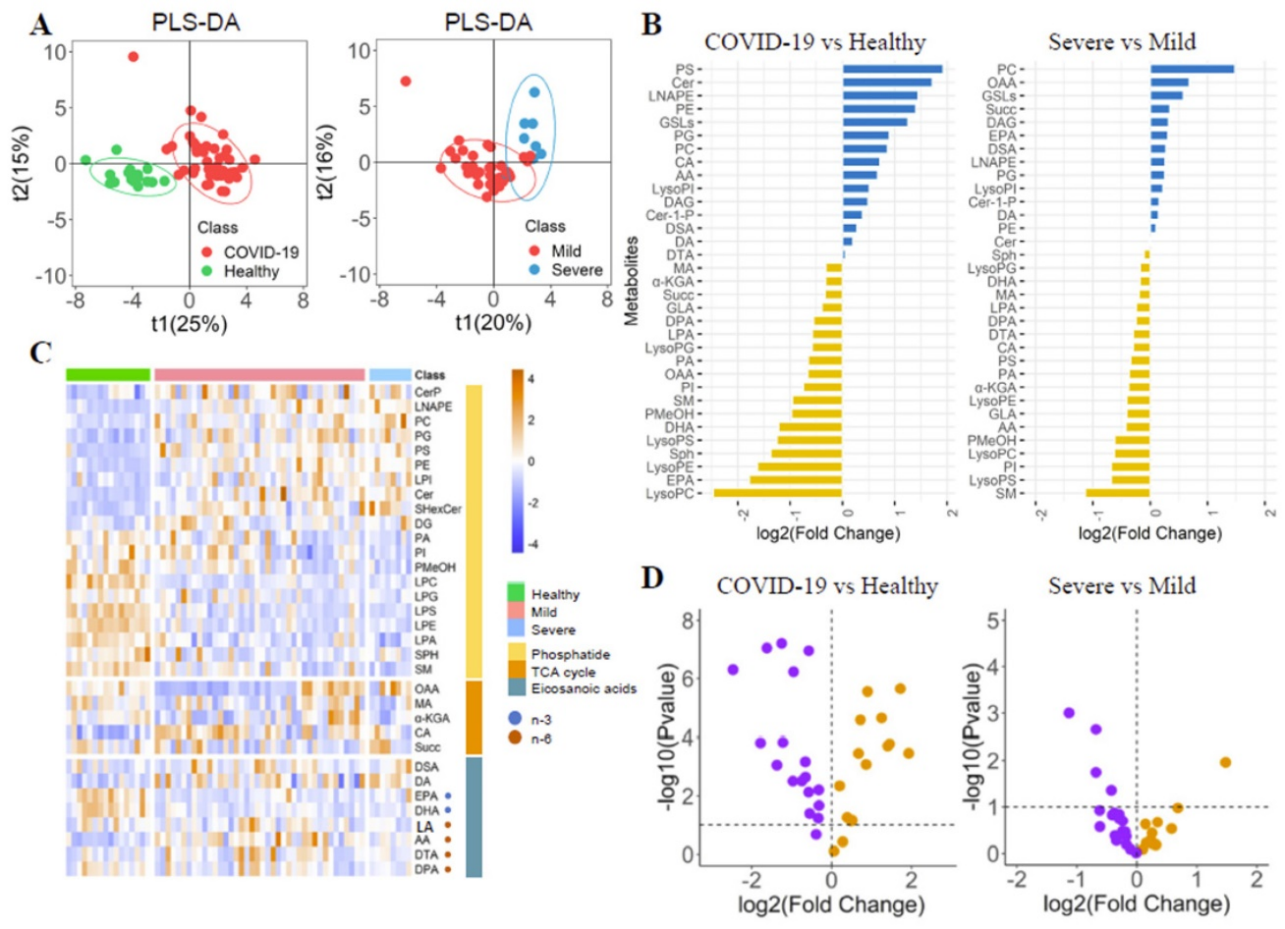
The Phospholipids, TCA cycle and Eicosanoic acids in COVID-19 patients. (A) PLS-DA score plots. (B) Boxplot of fold change (log10 scale) of all metabolites in COVID-19 patients and health control. (C) Heatmap of all metabolites in different groups. (D) Volcano plot of metabolites.

**Figure 2:**
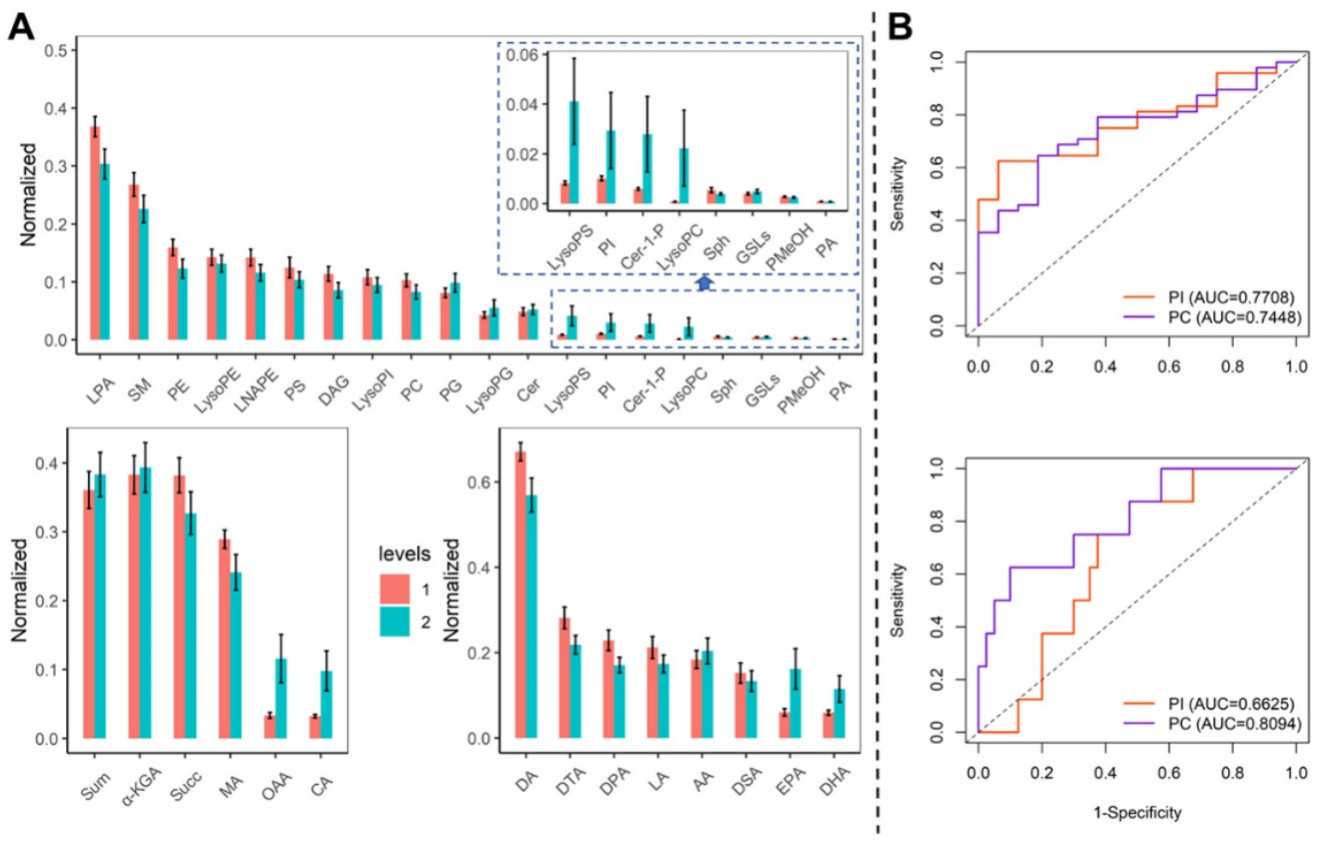
The trend of the diagnosis performance of phospholipid metabolisms. (A) Comparison of metabolites in blood samples on admission and before discharge. Levels 1: admission, 2: before discharge. (B) The ROC curve for PI and PA to identify COVID-19 patients from healthy controls (upper) and identify severe patients from mild patients (bottom).

The differences of phospholipid-related metabolites in COVID-19 patients were explored by PLS-DA (Figure 1A). Each metabolite's fold change (FC) was calculated (Figure 2B, Supplementary Table 1). The metabolite's heatmap (Figure 1C) and a volcano plot (Figure 1D) were shown. The phosphatidylethanolamine (PE) and phosphatidylcholine (PC) were significantly higher in COVID-19 patients than in healthy controls (log2(FC)=1.40 and 0.99, respectively, *P*<0.05). The severe group had a higher PI than the mild group (log2(FC)=1.19, P<0.05), but there was no significant difference in PE between the two groups. The opposite trend was observed for phosphatidylinositol (PI) (log2(FC)=-0.75 and -0.68 for healthy-patient and mild-severe, respectively, *P*<0.01). The three corresponding lysophosphatidic acids (LysoPE, LysoPC, and LysoPI) were found to be significantly lower, lower, and higher in patients than in healthy subjects (log2(FC)=-1.62, -2.46 and 0.51, respectively, *P*<0.05), but there were no significant differences between severe and mild patients. It could be seen that the trend of phosphatidic acid and the corresponding lysophosphatidic acid were not completely consistent. The ceramide (Cer), glycosphingolipids (GSLs), phosphatidylglycerol (PG) and phosphatidylserine (PS) were also found to be higher in COVID-19 patients (log2(FC)=1.72, 1.24, 0.90 and 1.93, respectively, *P*<0.05), while the phosphatidic acid (LPA), sphingosine (Sph) and phosphatidyl ethanol (PMeOH) were found to be lower in COVID-19 patients (log2(FC)=-0.65, -1.36 and -0.97, respectively, *P*<0.05). There were no significant differences between severe patients and mild patients for all of them.

The n-6 metabolite arachidonic acid (AA) in the eicosanoid pathway was significantly elevated in COVID-19 patients and severe patients (log2(FC)=0.67 and -0.42, respectively, *P*<0.05). Patients had lower levels of n-3 metabolites such as docosapentaenoic acid (DPA), eicosapentaenoic acid (EPA), and Docosahexaenoic acid (DHA) than healthy controls (log2(FC)=-0.55, -1.78, and -1.22, respectively, *P*<0.05). The patients had higher docosanoic acid (DA) than healthy controls (log2(FC)=0.19, *P*<0.05).

The levels of oxaloacetate (OAA), succinic acid (Succ), malic acid (MA), and α-ketoglutarate (α-KGA) in the TCA circulatory pathway were significantly lower in the patient than in healthy controls (log2(FC)=-0.66, -0.33, -0.32 and -0.32, respectively, *P*<0.05), but there was no significant difference between the mild and severe patients. Isocitric acid (CA) levels in patients were significantly higher than that in healthy controls (log2(FC)=0.71, *P*<0.05). Because the TCA cycle is a circular pathway, we believe that the difference in overall value can more clearly show the degree of energy difference, so we calculated the difference in overall value. The overall value was higher in COVID-19 patients (log2(FC)=0.33, *P*<0.05). Trends and diagnostic efficacy of phospholipid pathway-related proteins were also analyzed (Supplementary Figure 1).

We compared the metabolites in blood samples on admission and before discharge. The LPA, SM, PE, and PC were decreased, while the PI, LysoPG, LysoPC, and LysoPI were elevated (Figure 2A). The diagnosis of PI and PC was evaluated by the ROC curve (Figure 2B). The PI could identify the COVID-19 and severe patients with the AUC of 0.771 (95% CI: 0.656-0.886) and 0.663 (95% CI: 0.595-0.730). The PC could identify the COVID-19 patients and severe patients with the AUC of 0.775 (95% CI: 0.618-0.871) and 0.809 (95% CI: 0.638-0.981).

**Figure 3:**
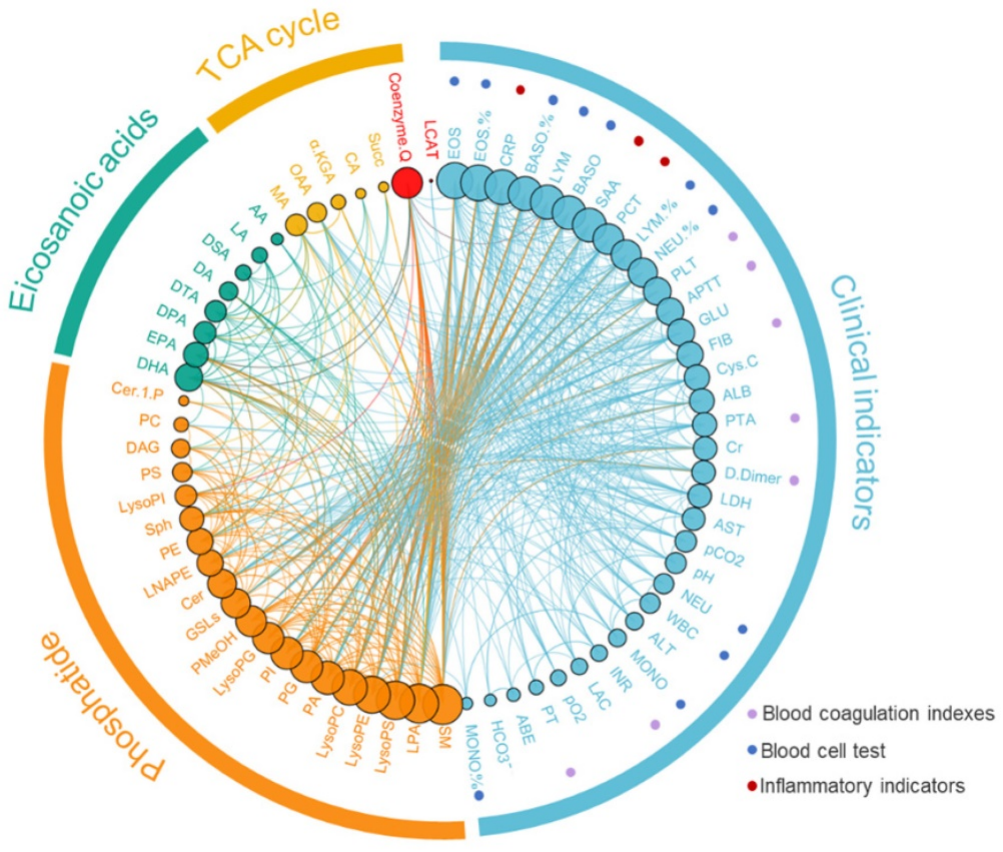
Correlation analysis of metabolomics and clinical indicators (Supplementary Table 2)

We analyzed the correlation between phospholipid metabolic pathway, energy metabolic pathway, inflammatory related metabolic pathway, and clinical indices (Figure 3, Supplementary Table 2). The results showed that the Cer was negatively correlated with the Sph and the SM (r=-0.297 and -0.358, *P*<0.001), and the Cer was positively correlated with the AA (r=0.573, *P*<0.001). The AA was negatively correlated with DHA and EPA (r=0.407 and 0.424, *P*<0.001). The D-dimmer was negatively correlated with lysophospholipid metabolites, including LysoPE, LysoPC, and LPA (r=-0.468, -0.512 and -0.335, *P*<0.001). The DAG was positively correlated with the total value of energy metabolism (r=0.235, *P*<0.05).

To further investigate the association of the upstream metabolites LysoPC and LysoPE with the downstream metabolite LPA, patients with D-dimmer >900 U/ml were identified as a high-risk group for thrombosis 19, 20. The results showed that LysoPC and LysoPE were found to be significantly higher in COVID-19 patients in the high-risk group for thrombosis than in patients with D-dimmer ≤900 U/ml (*P*<0.05), whereas LPA levels were lower (*P*<0.05). PLTP (phospholipid transporter), which transports phospholipid metabolites, also showed a downward trend in patients. The results showed that PLTP was significantly correlated with PE (r=-0.311, P<0.05).

## 4. Discussion

This study analyzed the trends and correlations of phospholipid metabolic pathways, PUFA (polyunsaturated fatty acid) inflammatory pathways and TCA circulating pathways in COVID-19 patients. PI and PC in the phospholipid metabolic pathway were found to distinguish between healthy people and COVID-19 patients and associated with the risk of exacerbation in patients. The results demonstrated that most phospholipid metabolites were significantly altered in patients, with PI and PC not only being able to distinguish between healthy individuals and COVID-19 patients but also be associated with disease severity. In addition, combined with the clinical characterization of the patients, we established Cer-SM, LPs and membrane-function related phospholipid pathways, which are associated with cellular pyrogenesis, thrombus formation and inflammation in the progression mechanism of COVID-19. Thus, the mechanism of the COVID-19 was explored from a metabolic perspective.

### 4.1 Molecular flow of phospholipid metabolites

**Figure 4:**
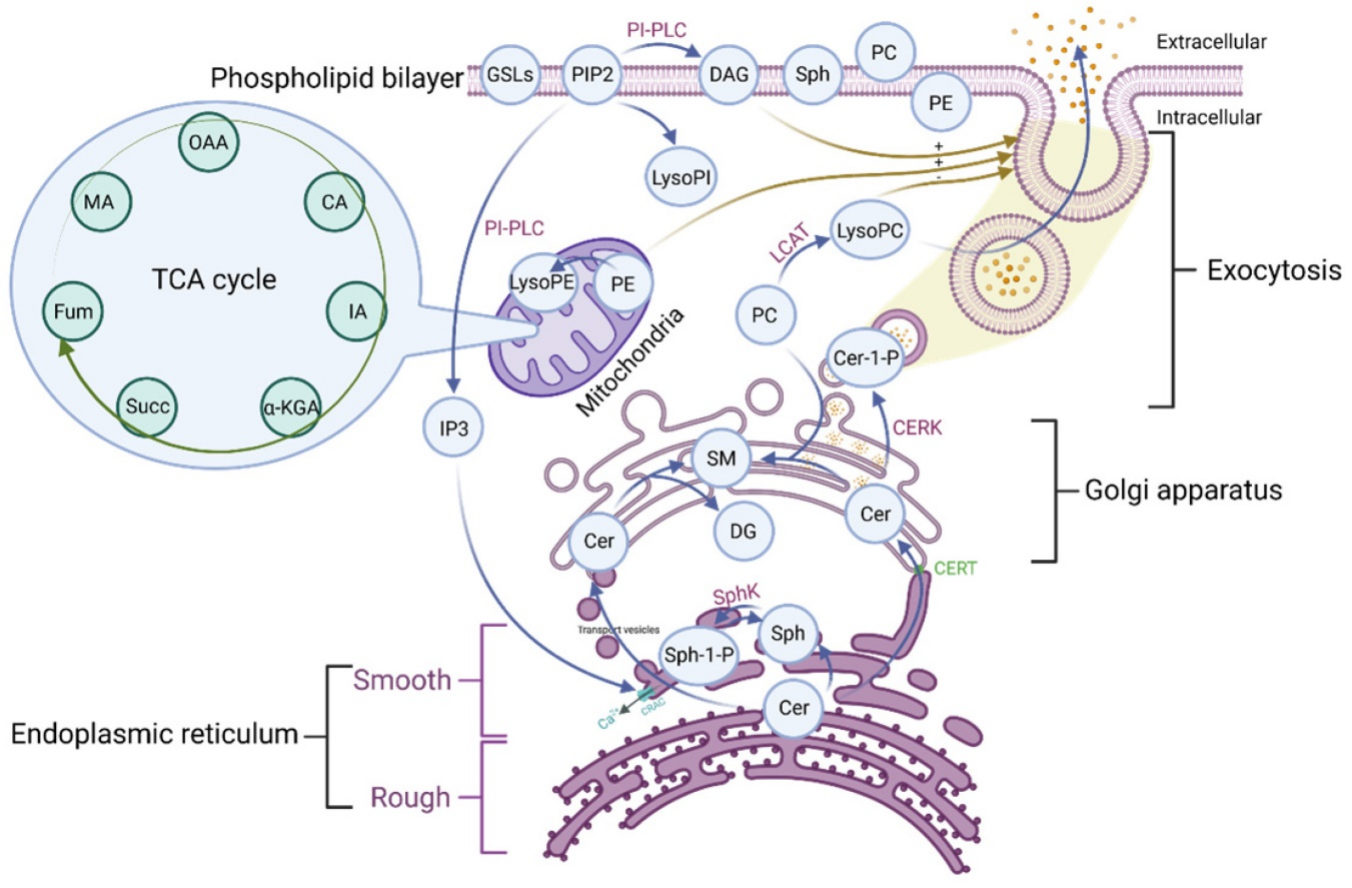
The pathway of the phospholipid and TCA. OAA: Oxaloacetate acid; CA: Citric acid; α-KGA: α-Ketoglutara acid; Succ: succinate acid; MA: Malate acid; GSLs: Glycosphingolip-ids; PIP2: Phosphatidylinositol-4, 5-diphosphate; LysoPI: Lysophosphatidylinositol; PI-PLC: Phosphoinositol specific phospholipase C; DAG: Diacylglycerol; Sph: Sphingosine; PC: Phosphatidylcholine; PE: Phosphatidylethanolamine; LysoPE: Lysophosphatidylethanolamine; LysoPC: Lysophosphatidylcholine; LCAT: Lecithin cholesterol lipoyl transferase; Cer: Ceramide; Cer-1-P: Ceramide 1-phosphate; SM: Sphingomyelin; DAG: Diacylglycerol; Sph-1-P: Sphingosine 1-phosphate; SphK: Sphingomyelin kinase; CERT: Ceramide transfer protein; CRAC: Endoplasmic reticulum Ca^2+^ release activates Ca^2+^ channels; IP3: Inositol 1,4,5-triphosphate; TCA: tricarboxylic acid cycle.

Phospholipid metabolic cycle could be seen in the life activities of various cells, including the TCA cycle, damage repair, signal transduction, inflammation induction, hemolysis and coagulation, and other effects 12, 14. COVID-19 was a disease that primarily affects the lungs and the amplification effect of subsequent inflammatory damage could cause systemic multisystem dysfunction 21. Most phospholipid-related metabolites could be seen as a significant trend in the heat map. Phospholipid function was very intricate. The establishment of the phospholipid acid pathway based on molecular structure and flow direction was a common analytical method that could intuitively reflect the transformation of phospholipid metabolites and their specific position in the cell, allowing the corresponding function to be speculated (**Figure 4**).

The majority of the Cer was formed in the rough endoplasmic reticulum before being transported to the Golgi apparatus. This pathway involves vesicular and non-vesicular transport, with the contact pattern (non-vesicular) being more common 22. Cer was turned into SM and DAG at the Golgi, and the majority of the remaining Cer was transformed into GSLs, which were critical for maintaining cell membrane stability 22. This specific region of tight adherence, which acts as one of the major barriers to non-vesicular transport structural domains, may contribute to the formation of phospholipid metabolite gradient concentrations 22. A small percentage of rest Cer was phosphorylated by the Cer kinase (CERK) to form CER-1-P 22. Cer could also be degraded by ceramidase to Sph and then phosphorylated to SPH-1-P by SphK. In the presence of phosphoinositol diphosphate specific phospholipase C, PIP2 (phosphatidylinositol bisphosphate) hydrolyzed into soluble IP3 (inositol triphosphate) and entered the cell. Meanwhile, the accompanying product DAG entered the membrane 23. Additionally, IP3 could rapidly diffuse into the cytosol, activate the CRAC (calcium-release-activated calcium channel) on the endoplasmic reticulum, and open the endoplasmic reticulum calcium pool. PIP2 could also be converted to LysoPI. LCAT (Lecithin cholesterol ester acyltransferase) was released by the liver and acted as a catalyst in plasma, converting lecithin PC into LysoPC and cholesterol lipids. PE is mainly located in the inner membrane, and its content is second only to PC. PE could be transformed into LysoPE, which is mainly located in the mitochondrial membrane 24. Therefore, there were many phospholipid metabolites and the overlapping effect of paths could also occur in the flow process. It was not easy to map each of these metabolites to clinical information in conventional molecular research. We grouped metabolites with the same pathway and similar functions and discussed them systematically concerning the specific clinical features of COVID-19.

### 4.2 Cer-SM cycle

Cer was significantly elevated in COVID-19 patients compared to healthy individuals in this study. As one of the main components of the cell membrane, Cer could be transformed into CER-1-P, Sph, and SM. Cer played a key role in programmed cell death induced by oxidative stress, inflammation, infection, and cycle arrest 25. Similarly, Sph, CER-1-P, and SM could regulate programmed cell death and promote cell survival 26. Sph and SM results showed opposite trends to Cer, showing a significant negative correlation. The formation and hydrolysis of SM and Cer are reversible 27. Kitatani et al indicated that the hydrolysis of SM and the increase of Cer level in the SM-Cer cycle would lead to periodic cell stasis or cell pyrosis in 2015 28. SARS-CoV-2 activates inflammatory bodies and induces cell lysis and death 29. The comparison with CRP showed that the pyroptosis effect increased with the progression of inflammatory infiltration 30. The study also found a positive correlation between Cer and CRP. Haimovitz et al. found that Cer could amplify pyroptosis signals in infected patients 31. Therefore, it was reasonable to conclude that lung injury caused by inflammation and pyroptosis due to SARS-COV-2 invasion is closely associated with the trend of elevated Cer levels in the Cer-SM cycle.

Furthermore, in the analysis of anti-inflammatory n-3 and pro-inflammatory n-6 of the PUFA inflammatory metabolic pathway, we also found increased levels of the n-6 core (metabolite arachidonic acid) and decreased levels of the n-3 metabolites (DHA and EPA). Arachidonic acid was positively correlated with Cer, while DHA and EPA were negatively correlated with Cer. These results further provided evidence that Cer, which corresponds to an overactive immune response, plays an important role in promoting the exacerbation of COVID-19 and even triggering inflammatory storms.

### 4.3 LPs pathway

After SARS-CoV-2 infection, endothelial injury, complement elevation, hypoxia stress, and many other factors led to blood flow hypercoagulation. Reduced pulmonary perfusion with microcirculation obstruction, which significantly affects ventilatory function, could precede ARDS and was one of the main causes of poor prognosis in severe patients 32, 33. It was necessary to explore the role of Lysophospholipids in the coagulation mechanism. In the study, LysoPC, LysoPI, and LysoPE all belonged to Lysophospholipids, which had the function of dissolving cell membrane to promote fibrinolytic. LysoPC, LysoPI, and LysoPE could transform LPA, an intercellular signaling substance that promoted coagulation progress. LPA also showed a downward trend in patients. LPA could accelerate platelet aggregation and activate platelet to release LPA further, thus creating a positive feedback effect 34. LPA levels in COVID-19 patients in the study were down-regulated to alleviate hypercoagulability. However, the results revealed that LysoPE and LysoPC were also significantly decreased in patients. The trend changes of downstream LPA and upstream LysoPC and LysoPE were consistent in metabolic pathways, but they have opposite functions. Were the results of upstream and downstream metabolites contradictory? Borghi et al. pointed out that 57% of COVID-19 patients tend to have thrombosis, but AT/APTT was abnormally prolonged 35. However, we believed that the pathway mechanism cannot be analyzed solely based on the function of individual indicators. The potential mechanism could still be found and speculated by combining the changes of metabolites upstream and downstream of the metabolic pathway. However, index contradictions are not uncommon in the analysis process.

To further clarify the association between upstream LysoPC, LysoPE and downstream LPA, we conducted a comparative analysis between them and D-dimmer. The popular fibrinolytic protein D-dimmer was a characteristic indicator for evaluating COVID-19 coagulation abnormalities 36. Endothelial damage caused by the inflammatory effects of SARS-CoV-2 invasion leads to thrombotic tendencies, requiring an increase in D-dimmer to maintain healthy blood flow 37. The fibrinolytic effect of D-dimmer was similar to those of LysoPC and LysoPE, and they were statistically correlated. Adopting D-dimmer =900 U/mL as the grouping boundary for risk of thrombosis (Higher than 900U/mL indicated a high risk), we found that LysoPC and LysoPE were significantly increased in the D-dimmer >900 U/mL than in the D-dimmer ≤900 U/mL groups of COVID-19 patients. Nevertheless, LPA levels were lower in the D-dimmer ≤900 U/mL groups. Patients with severe COVID-19 are in coagulation or fibrinolytic state, determining the trend of coagulation indicators for different functions. We established the following hypothesis based on the results: Downstream LPA could be regulated by upstream LysoPC and LysoPE. Changes in the lysophospholipid pathway could reflect diverse biological functions 38. The establishment of upstream and downstream concentration gradients was the basis of biological function. LPA was defined as regulatory material. LPA decrease was required during thrombogenesis to reduce blood's tendency to hypercoagulability. Thus, a decrease in upstream LysoPC/LysoPE led to a decrease in downstream LPA transformation in response to the progression of hypercoagulability.

In the D-dimmer>900 U/mL stage, the risk of DIC increased. The decrease in fibrinogen and platelet in this group showed excessive consumption of clotting substances, and the fibrinolysis process was enhanced proportionally. LysoPC/LysoPE increased in response to micro thrombosis, but LPA levels were lower. The reason for this is that upstream LPs regulate LPA. Activation signals generated by platelet depletion are reduced, leading to inhibition of LPA production.

### 4.4 Membrane-function related phospholipid pathway

Phospholipid metabolism also played an important role in maintaining the physiological state of the cytomembrane and organelle membrane. Changes in membrane morphology and attachment channels were necessary for granulocyte migration, erythrocyte deformation, phagocytosis, secretion, adhesion, and contact signal transduction 39. PI, PC, and PE were the most common membrane-structure-related phospholipid metabolites. The study showed a significant increase in PE and PC levels. PE contributes to membrane protein folding, protects against SARS-CoV-2-induced pyroptosis and stress, maintains respiratory chain complex activity, and initiates autophagy to fight inflammation and infection 24. In a COVID-19 study, elevated PC was considered to play a role in mediating phagocytosis of neutrophils and macrophages, which are important links in the initiation and maintenance of immunity 12. This immunophagocytic effect was an energy consumption process. It could be seen in the study that the overall active degree of energy cycle (TCA cycle) in severe patients in the resting state was higher than that in the mild group. Patients' higher energy metabolism level at the time of admission indicated the body was under stress, maintaining energy consumption processes such as fever and breathlessness. It was important to note that inflammatory storms did not always lead to hyperimmunity. In the study, three severe COVID-19 patients with sepsis showed a downward trend in their energy cycle activity compared to the mild group, even lower than the healthy people, which might be related to immunosuppression caused by the inflammatory storm. PC and PE were negatively correlated with n-3 of DHA and EPA. As anti-inflammatory feedback indicators, DHA and EPA showed a downward trend in the study, indicating that the internal environment was developing towards a pro-inflammatory trend. PC and PE provided the structural basis for the activity of autoimmune-contributing cells.

In contrast to PC and PE, PI was lower in COVID-19 patients than in healthy subjects and was lower in patients with severe disease than in patients with mild disease. According to existing research results, PI could regulate membrane proteins' spatial structure, stabilize the contact sites of adjacent membranes for inter-cell ion exchange and signal transduction, and adjust the cytoskeleton to maintain normal cell life activities 40. The decrease of PI level reflected the imbalance of the microenvironment caused by the inflammatory storm in the severe stage of COVID-19, and the effect on PI was more of an inhibitory effect, which refuted the view that the breakdown of the cell membrane after lung injury led to the increase of phospholipid related metabolites. Our finding of the increased trend of PC and PE was consistent with a previous study 9. The analysis of the decrease in PI abnormally needs to be further explored from the molecular perspective in the future. The changes of DAG were consistent with PI, and DAG played an important role in the budding of membrane and the formation of microvesicles, which was closely related to the energy cycle 23. The results also showed a significant positive correlation between DAG and the energy cycle. We considered that the decrease in PI abnormality contrary to PC and PE might be related to the structure of the intracellular endoplasmic reticulum (ER), a complex membrane network that extended into the entire cytoplasm and formed stable contact with almost all organelles. Membrane properties establish phospholipid gradients at specific locations and corresponding metabolic pathways, so phospholipid metabolites trends were more significant than other metabolites 40.

### 4.5 PLTP

PLTP (Phospholipid transfer protein), which could transport phospholipid metabolites, also showed an abnormal decline trend in COVID-19 patients. The result showed that PLTP was significantly correlated with PE. In previous studies, PLTP was believed to have anti-inflammatory effects and reduce the risk of sepsis 41. Gautier et al. proposed the important value of PLTP in microenvironmental protection and innate immunity, and PLTP was also considered a potential therapeutic target for infection-related diseases 41, 42. Therefore, PLTP transported phospholipid metabolites of different functions, and the negative effects caused by the decrease of PLTP content are comprehensive. Invasion of SARS-CoV-2 inhibited the transport of PLTP and reduced the activity of the phospholipid metabolic pathway, thus promoting the progression of the disease.

## 5. Conclusion

In this study, we have established phospholipid metabolic pathways associated with the mechanism of COVID-19 progression based on the trends and specific functions of phospholipid metabolites, combined with the molecular field and clinical practice. The significance of phospholipid metabolic trends was analyzed in the context of TCA circulatory pathways and inflammatory metabolic pathways. In the Cer-SM cycle, we found that Cer was elevated while SM was decreased in COVID-19 patients, and the function of Cer in amplifying the pyroptosis signal may be related to the mechanism of lung injury. In LPs pathway analysis, we concluded that upstream LysoPC and LysoPE mainly regulated LPA and elevated LPA led to a high risk of COVID-19 thrombosis. Moreover, combining the TCA cycle and the n-3/n-6 pathway, we found that the common phospholipid metabolites, including PE, PI, and PC, played an important role in maintaining inflammatory cell activity. PI and PC could be used as metabolites to assess the risk of disease progression in COVID-19 patients.

## Supplementary Material

Supplementary figures and table legends.

Supplementary table 1.

Supplementary table 2.

## Figures and Tables

**Table 1:** The basic information of the control group and COVID-19 group

	Healthy Control	Mild	Severe	p
n	16	40	8	
Age	37.50 (29.00, 45.25)	43.00 (29.00, 51.50)	75.00 (70.00, 76.00)	<0.001
CRP, ng/ml	——	10.00 (10.00, 12.11)	13.33 (10.00, 59.98)	0.02
PCT, ng/ml	——	0.06 (0.05, 0.08)	0.10 (0.05, 0.26)	0.327
SAA, ng/ml	——	36.90 (8.68, 83.64)	91.65 (25.54, 213.56)	0.136
*** Blood cell detection***				
WBC, 10^9/L	6.36 (5.42, 6.69)	5.09 (4.44, 5.85)	6.31 (5.12, 7.89)	0.024
NEU, 10^9/L	3.59 (3.20, 4.22)	3.18 (2.39, 4.03)	4.88 (3.26, 6.87)	0.036
NEU%	60.20 (53.70, 62.10)	60.10 (48.90, 73.20)	76.70 (69.40, 80.20)	0.005
LYM, 10^9/L	1.84 (1.69, 2.36)	1.26 (0.89, 1.79)	1.07 (0.87, 1.11)	<0.001
LYM%	32.50 (28.20, 36.75)	25.10 (17.10, 38.50)	15.30 (11.60, 21.90)	0.003
MONO, 10^9/L	0.33 (0.28, 0.36)	0.44 (0.31, 0.56)	0.44 (0.35, 0.54)	0.039
MONO%	5.30 (4.45, 5.55)	8.60 (6.80, 10.20)	7.50 (4.40, 10.50)	0.001
BASO, 10^9/L	0.04 (0.02, 0.05)	0.01 (0.01, 0.02)	0.01 (0.00, 0.01)	<0.001
BASO%	0.70 (0.40, 0.80)	0.30 (0.20, 0.50)	0.10 (0.00, 0.20)	<0.001
EOS, 10^9/L	0.13 (0.07, 0.20)	0.02 (0.00, 0.11)	0.01 (0.00, 0.02)	<0.001
EOS%	2.10 (1.20, 3.25)	0.40 (0.00, 2.20)	0.10 (0.00, 0.30)	<0.001
*** Coagulation tests***				
PT, second	——	13.49 (12.54, 14.16)	12.95 (12.69, 13.27)	0.232
APTT, second	——	33.00 (25.14, 40.15)	35.85 (32.71, 38.88)	0.595
D-Dimer, ug/L	——	0.98 (0.28, 1090.00)	0.85 (0.35, 1010.00)	0.894
FIB, g/L	——	3.04 (2.70, 4.13)	3.26 (2.97, 3.81)	0.353
INR	——	1.06 (1.01, 1.14)	1.02 (1.00, 1.08)	0.242
PTA, %	——	85.00 (79.50, 93.58)	91.25 (83.94, 100.25)	0.326
PLT, 10^9/L	259.00 (219.00, 298.50)	195.00 (163.00, 264.00)	161.00 (134.00, 198.00)	0.002
*** Extra pulmonary organs***				
ALT, U/L	12.40 (9.45, 21.10)	16.00 (12.65, 19.95)	14.60 (13.10, 52.90)	0.267
AST, U/L	17.40 (15.05, 19.65)	16.50 (14.43, 18.92)	28.40 (14.20, 94.10)	0.521
LDH, U/L	——	174.00 (150.00, 197.00)	198.00 (180.50, 221.50)	0.161
GLU, mmol/L	——	5.30 (4.80, 6.95)	9.10 (7.73, 9.80)	0.013
Cr, mmol/L	50.00 (45.00, 55.35)	74.60 (51.55, 84.15)	89.10 (76.62, 93.40)	0.001
Cys-C, mg/L	——	0.85 (0.73, 1.10)	1.41 (1.17, 1.49)	0.009
*** Arterial blood gas analysis***				
PH	——	7.38 (7.33, 7.40)	7.42 (7.41, 7.44)	0.008
PCO_2_, mmHg	——	42.75 (36.65, 45.60)	37.40 (33.40, 39.38)	0.078
PO_2_, mmHg	——	91.75 (85.15, 105.75)	90.45 (77.30, 111.25)	0.695
HCO_3_^-^(P), mmol/L	——	25.00 (22.42, 26.63)	24.00 (22.27, 25.65)	0.784
LAC, mmol/L	——	1.70 (1.15, 2.45)	1.40 (1.22, 1.58)	0.339

CRP: C-reaction protein; PCT: Procalcitonin; SAA: Human serum amyloid A; WBC: White blood cell; NEU: Neutrophil; LYM: Lymphocyte; MONO: Monocyte; BASO: Basophil; EOS: Eosinophils; APTT: Activated partial thromboplastin time; PT: Prothrombin time; INR: International normalized ratio; FIB: Fibrinogen. CRP: C-reaction protein; PCT: Procalcitonin; SAA: Human serum amyloid A; WBC: White blood cell; NEU: Neutrophil; LYM: Lymphocyte; MONO: Monocyte; BASO: Basophil; EOS: Eosinophils; APTT: Activated partial thromboplastin time; PT: Prothrombin time; INR: International normalized ratio; FIB: Fibrinogen; PTA: Prothrombin time activity; PLT: Platelet; ALT: Alanine aminotransferase; AST: Aspartate aminotransferase; LDH: Lactate dehydrogenase; GLU: Glutamic acid; Cr: Creatinine; Cys-C: Cystatin C; LAC: Blood lactic acid.

## Abbreviations

CRP: C-reaction protein
PCT: Procalcitonin
SAA: Human serum amyloid A
WBC: White blood cell
NEU: Neutrophil
LYM: Lymphocyte
MONO: Monocyte
BASO: Basophil
EOS: Eosinophils
APTT: Activated partial thromboplastin time
PT: Prothrombin time
INR: International normalized ratio
FIB: Fibrinogen
PE: Phosphatidylethanolamine
PC: Phosphatidylcholine
PI: Phosphatidylinositol
PS: Phosphatidylserine
LysoPE: Lysophosphatidylethanolamine
LysoPC: Lysophosphatidylcholine
LysoPI: Lysophosphatidylinositol
OAA: Oxaloacetate acid
CA: Citric acid
α-KGA: α-Ketoglutara acid
Succ: Succinate acid
MA: Malate acid
GSLs: Glycosphingolipids
DAG: Diacylglycerol
Sph: Sphingosine
Cer: Ceramide
Cer-1-P: Ceramide 1-phosphate
SM: Sphingomyelin
DAG: Diacylglycerol
PmeOH: Phosphatidyl methanol
LPA: lysophosphatidic acid
DSA: Docosadienoic acid
DA: Docosanoic acid
EPA: Eicosapentaenoic acid
DHA: Docosahexaenoic acid
GLA: Linolenic acid
AA: Arachidonic acid
DTA: Docosatetraenoic acid
DPA: Docosapentaenoic acid
Sph-1-P: Sphingosine 1-phosphate
SphK: Sphingomyelin kinase
S1P: Sphingosine-1-phosphate
CERT: Ceramide transfer protein
LPs: Lysophospholipid
PIP2: Phosphatidylinositol-4, 5-diphosphate
PI-PLC: Phosphoinositol specific phospholipase C
LCAT: Lecithin cholesterol lipoyl transferase
CRAC: Endoplasmic reticulum Ca^2+^ release activates Ca^2+^ channels
IP3: Inositol 1,4,5-triphosphate
TCA: tricarboxylic acid cycle
LNAPE: N-acyl-lysophosphatidylethanolamine
DIC: Disseminated intravascular coagulation
PLTP: Phospholipid transfer protein
