# Supplementary material for: Effect of a Functional Phospholipid Metabolome-Protein Association Pathway on the Mechanism of COVID-19 Disease Progression
Source: Int J Biol Sci. 2022 Jul 11;18(12):4618–28. doi: 10.7150/ijbs.72450 (PMC9305269; doi:10.7150/ijbs.72450)
Supplement: Supplementary file 1 — Supplementary figures and table legends. [file ijbsv18p4618s1.pdf]

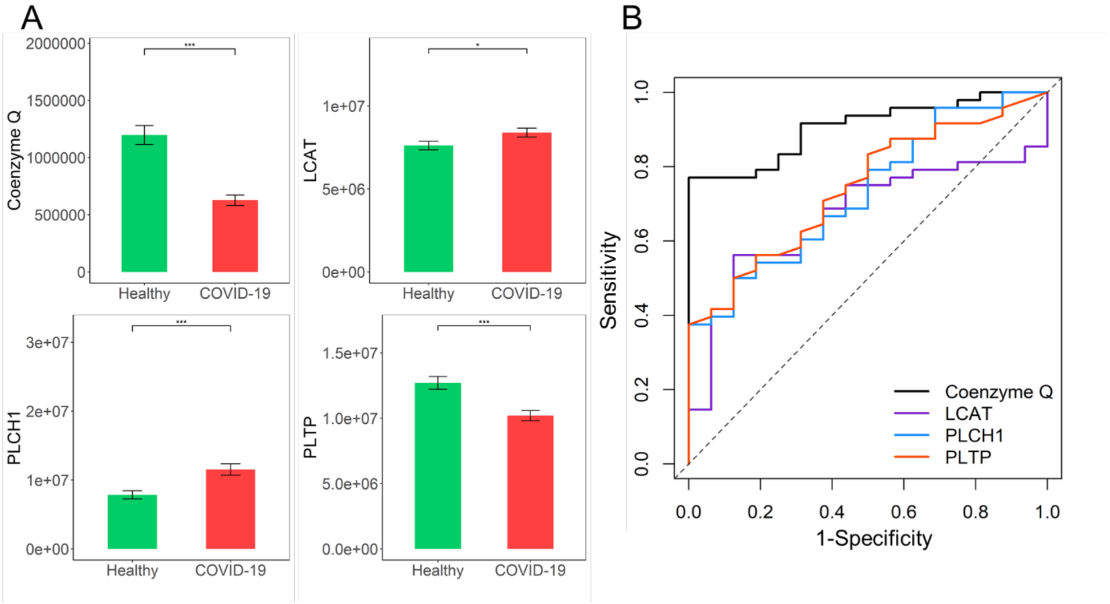


Figure S1. Trends and diagnostic efficacy of phospholipid pathway related proteins. (A) The comparison of the proteins between healthy controls and COVID-19 patients. (B) The diagnosis of the proteins.
